# A Neuro-Mechanical Model of a Single Leg Joint Highlighting the Basic Physiological Role of Fast and Slow Muscle Fibres of an Insect Muscle System

**DOI:** 10.1371/journal.pone.0078247

**Published:** 2013-11-11

**Authors:** Tibor Istvan Toth, Joachim Schmidt, Ansgar Büschges, Silvia Daun-Gruhn

**Affiliations:** 1 Emmy Noether Research Group of Computational Biology, Department of Animal Physiology, University of Cologne, Cologne, Germany; 2 Department of Animal Physiology, University of Cologne, Cologne, Germany; Mount Sinai School of Medicine, United States of America

## Abstract

In legged animals, the muscle system has a dual function: to produce forces and torques necessary to move the limbs in a systematic way, and to maintain the body in a static position. These two functions are performed by the contribution of specialized motor units, i.e. motoneurons driving sets of specialized muscle fibres. With reference to their overall contraction and metabolic properties they are called fast and slow muscle fibres and can be found ubiquitously in skeletal muscles. Both fibre types are active during stepping, but only the slow ones maintain the posture of the body. From these findings, the general hypothesis on a functional segregation between both fibre types and their neuronal control has arisen. Earlier muscle models did not fully take this aspect into account. They either focused on certain aspects of muscular function or were developed to describe specific behaviours only. By contrast, our neuro-mechanical model is more general as it allows functionally to differentiate between static and dynamic aspects of movement control. It does so by including both muscle fibre types and separate motoneuron drives. Our model helps to gain a deeper insight into how the nervous system might combine neuronal control of locomotion and posture. It predicts that (1) positioning the leg at a specific retraction angle in steady state is most likely due to the extent of recruitment of slow muscle fibres and not to the force developed in the individual fibres of the antagonistic muscles; (2) the fast muscle fibres of antagonistic muscles contract alternately during stepping, while co-contraction of the slow muscle fibres takes place during steady state; (3) there are several possible ways of transition between movement and steady state of the leg achieved by varying the time course of recruitment of the fibres in the participating muscles.

## Introduction

In legged animals, the muscles of the limbs have two basic functions: i) to produce forces and torques in order to move the limbs of the body during fast and slow locomotion and other rhythmic or episodic behaviours, and ii) to hold the body or its parts in a static position (posture). In order to perform these tasks effectively, muscle fibres of differing contraction dynamics evolved in many species [1]. A muscle can be subdivided into motor units: a motoneuron (MN) and all of the muscle fibres it innervates. In mammals, the fibres of a motor unit show similar biochemical and histochemical properties (for review see [2].) By contrast, in arthropods, a single muscle fibre may be innervated by more than one MN, and the fibres innervated by one MN may exhibit different electrophysiological, histochemical and contractile properties (e.g. [3]–[7]). These properties may allow for fine tuned movements despite the low number of motoneurons that innervate an arthropod muscle. Motor units are usually recruited in the order of decreasing electrical input resistance (size principle, [8], [9]), but this can be overruled (for reviews see [10], [11]).

In the stick insect, for example, slow and fast muscle fibres were discovered in the extensor tibiae muscle. Moreover, they were found to be anatomically (spatially) separated [7], [12]. It was also shown by [12] that the slow and fast fibres have different functional roles: both are active during stepping (locomotion) while only the former are active during maintaining the posture of the animal. The MNs innervating the fast fibres are normally not active during static positions of the stick insect. In addition, there exist common inhibitory MNs, which simultaneously inhibit the slow leg muscles during movement. In particular, the common inhibitory MN, denoted CI1, inhibits the slow muscle fibres of all main leg muscles, except for the m. flexor tibiae, which is inhibited by two different MNs CI2 and CI3. Up to recently, the presence of the differentiation between muscle fibres could only be confirmed with certainty in the extensor muscle. However, there is now experimental evidence that slow and fast muscle fibres are present in all three main muscle pairs (protractor-retractor, levator-depressor, and extensor-flexor) of the leg [13]. It therefore makes sense to use this property in models describing the neuronal control of muscle systems in insects.

In an earlier work [14], we constructed a neuro-mechanical model of the protractor-retractor muscle system, based on experimental data from the stick insect. This model included a central pattern generator (CPG), MNs that innervate the protractor and the retractor muscles, and interneurons (INs) connecting the CPGs to the MNs. It also comprised the models of the muscles just mentioned. The neurons of the CPG and the INs were non-spiking, and were described by the same, simple Hodgkin-Huxley-type model [15] but, of course, with different values of the model parameters. The MNs were also described by a Hodgkin-Huxley-type model but they could fire proper action potentials. We provide the model equations in Appendix S1. The model of the muscles was derived from Hill's model [16] with strong simplifications. It will be briefly described in the Methods below. This model is extended here to include slow muscle fibres beside the fast ones. The extension has been motivated by experimental results in which the leg, the femur in particular, showed only small-amplitude angular movements, or was held in a static horizontal position, for instance, during sideward stepping. Experimental findings (e.g. [7], [12]) clearly proved that slow muscle fibres were necessary to carry out the tasks just mentioned. We also found that our original model, with only the fast muscle fibres, could not produce these types of behaviour. The inclusion of slow muscle fibres is to help remedy this shortcoming. For the same reason, gradual recruitment of the muscle fibres is also implemented in the present, extended version. This new property enables the model to produce arbitrary static angles at the thorax-coxa joint of the leg. Also, it increases the overall flexibility of the model. However, we do not take the variability of the electrophysiological properties of slow and fast MNs into account. Nor do we include, at this stage, the common inhibitor MNs in the model, and thus neglect the residual stiffness, which is discernible in the slow muscle fibres. The reason for this is to keep the model extension as simple as possible in order to be able to concentrate on the direct and specific effects produced by the slow and fast muscle fibre types. In the accompanying paper (Toth et al., unpublished results) in which we shall consider a model with both slow and fast muscle fibres in all main leg muscles and their recruitment implemented, we shall also investigate the effects of residual stiffness of the slow muscle fibres and of the common inhibitory MNs. We shall make use of them to elucidate the detailed mechanism of stop and start of the stepping movement (locomotion). In present paper, we report the results which relate to the basic functions of the slow and fast muscle fibres. Important points in the simulations are the presence or absence of co-contraction of antagonistic muscles, and the co-activation, or the lack of it, of the MNs driving them. The model makes predictions related to these points. We can, by means of the model, show how recruitment proves to be an effective mechanism to achieve and maintain (near) steady-state positions, and also during transition from steady state to angular movements.

## Methods: The extended model of the protractor-retractor muscle system

**Figure 1:**
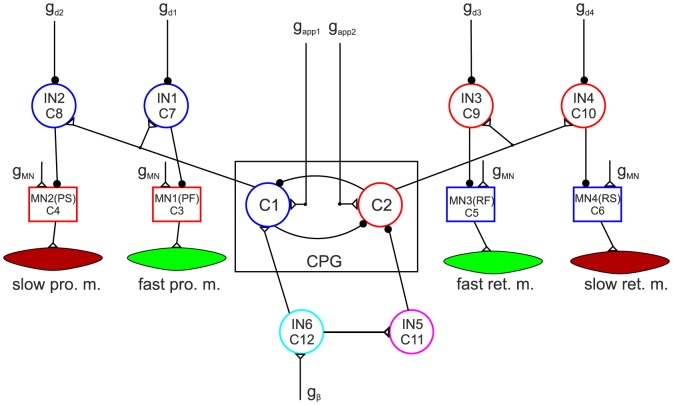
The extended model of the protractor-retractor muscle system. The model consists of a central pattern generator: CPG, slow and fast protractor and retractor muscles as indicated (slow pro. m. etc.), the corresponding motoneurons: MN(PS) etc., 4 inhibitory interneurons (IN1–IN4) connecting the CPG to the motoneurons, and two additional interneurons (IN5–IN6), which convey neuronal signals to the CPG from sense organs of other joints of the same leg, or possibly of other legs. 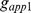, 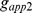 are conductances of the driving currents to C1 and C2, respectively. 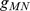 is the conductance of the common (central) input current to all motoneurons. 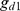
–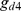 are conductances of the inhibitory currents to IN1–IN4, respectively. 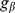 is the conductance of the sensory input current from the levator-depressor muscle system. Empty triangles are excitatory synapses; filled black circles on neurons are inhibitory synapses. The tiny black circles on synaptic paths are branching points.

**Figure 2:**
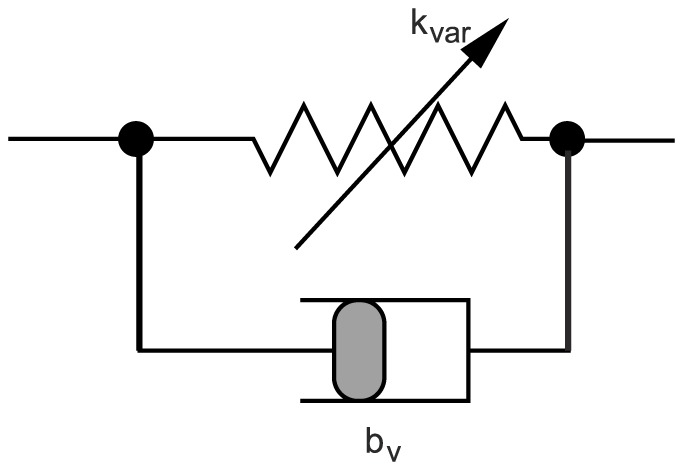
Schematic picture of the simplified muscle model. 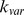
: variable spring constant (elasticity modulus). The actual value of 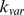 is determined by the activity of the connecting motoneuron; 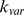 can be regarded as (actual) contractility of the muscle. 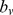: coefficient of the damping produced by viscosity. The torque generated by the damping is assumed to be proportional to the contraction velocity of the muscle fibre with a constant value of 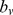 for a given muscle type.

Fig. 1 shows the network of the extended model. Its components are briefly described in the figure legend, but a more detailed description of them can be found in [14]. Its most important new property is that it now comprises slow muscle fibres together with their driving MNs, and inhibitory INs that connect the MNs to the CPG. As in the earlier version, the INs convey rhythmic inhibition from the CPG to the MNs (to both fast and slow.) The rhythmic inhibition can be modified or even abolished by increasing or decreasing the (central) inhibition to the INs (by changing the corresponding conductance 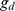 of the inhibitory current). The slow and fast muscle fibres differ by the dynamics of their response to neuronal excitation from the innervating MN. Thus the fast muscle fibres have fast response dynamics and the slow ones much slower response dynamics. The muscles are modelled as nonlinear springs [17] with variable spring constant (stiffness) and damping (viscosity) unit, as shown in Fig. 2. This is a gross simplification of Hill's muscle model [16]. In particular, our simplified model does not have explicit passive elements.

We also strongly simplified the force-velocity relationship of Hill's model by making it linear, assuming that the velocity range of the movements remains sufficiently narrow, as in the case of stick insects. The equation of mechanical motion of the femur under the influence of the (slow and fast) protractor and retractor muscles is given in Appendix S2. The elastic properties of the muscle fibres, as expressed by their spring constants, are identical. To be more specific, we recall the equations of the muscle model used in [14].
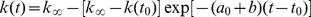
(1)

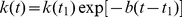
(2)Here, 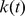 is the value of the spring constant at time 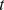, 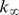 is its stationary (end) value. Eqn. 1 is valid during a MN action potential, and eqn. 2 otherwise. Note that the spring constant will eventually vanish if the activity of the innervating MN ceases. However, at switching the direction of movement of the femur, vanishing angular velocity and acceleration are assumed. These conditions impose constraints on the stationary values (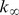) of the spring constants. As a consequence, the spring constants can only vanish, if *neither* of the muscles of the antagonistic muscle pair receives neuronal drive from its MN (for a detailed explanation see [14]). Both fast and slow muscle fibres have been modelled this way. The only difference between the two muscle types therefore is their differing response dynamics.

The rate constants in eqn.s 1 and 2 are 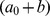 and 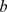, respectively, the former being much larger than the latter. These parameters characterize the kinetics of the muscle contraction. Slow muscle fibres have therefore small rate constants (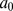 values). Specifically, during retraction, the fast retractor muscle fibres have 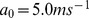, and the protractor ones 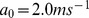. During protraction, the rate constant of the fast retractor muscle fibres is 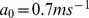, and that of the fast protractor muscle fibres 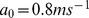. The values of the corresponding rate constants of the slow muscle fibres have been chosen to be 100 times smaller (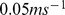 etc.). The relaxation rate constants (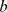 values) have identical values in both muscle types (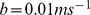 for all muscle types). These choices ensure that the dynamics of the angular movement match that seen in the experiments. For further details, see [14].

### Implementing muscle recruitment

So far, all muscles, fast or slow, have been regarded as “large”, single muscle fibres that were either activated, i.e. recruited, or not. However, all muscles that are included in the model consist of several motor units, not all of which need to be activated during muscle activity in order to carry out a movement, or to maintain the spatial position, of an extremity. Moreover, partial recruitment of muscle fibres is an important tool for gradually increasing or decreasing the total muscle force. It is therefore important to take account of this fact in the model, too. The ability of gradual recruitment of the muscle fibres increases the flexibility of our muscle model. This flexibility will indeed be needed to simulate various static or dynamic conditions produced by the muscle activity.

**Figure 3:**
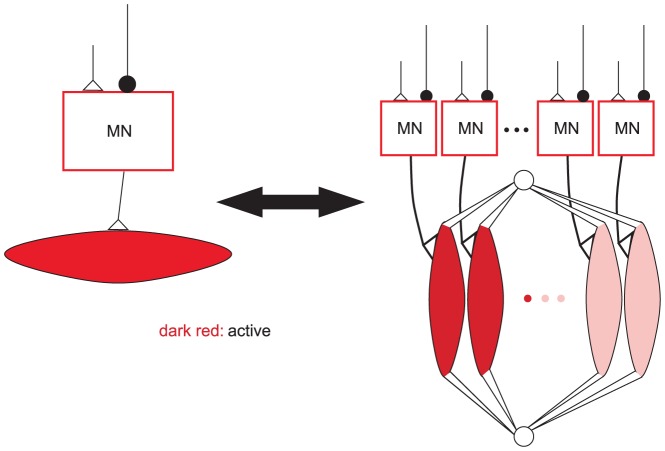
Modelling of recruitment. The single motor unit is symbolically replaced by a number of identical motor units in the model. The motoneurons are identical and the activated ones are synchronously driven. The mechanical and kinetic properties of the muscle fibres arranged in parallel are also identical for all fibres. Dark red fibres represent the recruited, i.e. active muscle fibres.

The implementation of recruitment in the model is schematically illustrated in Fig. 3. The main idea is that a MN-muscle functional unit of a given type (slow or fast) in Fig. 1, now represents a number of motor units. The MNs of these units have identical properties and, if some of them are activated, it is done synchronously. That is the MN in Fig. 1 exhibits activity as long as at least one motor unit is active. The muscles in Fig. 1 are also thought to consist of several fibres belonging to different motor units of the same type. The mechanical and kinetic properties of the fibres are assumed to be identical. The only difference between them is that, at a given instant of time, some of them are activated, i.e. recruited, others are not. The quantitative formulation of the effect of recruitment is very simple: a linearly proportional relationship between muscle contractility and the proportion of recruited fibres in the whole muscle. That is,
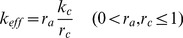
(3)where 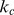 is the total value of the spring constant of all recruited fibres of the muscle in “control conditions”, which we take to be normal stepping. The quantities 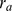 and 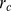 are the proportions of the actually recruited muscle fibres and those in control conditions, respectively. Finally, 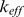 is the effective (actual) total value of the spring constant of the muscle, which determines its actual contractility. Here, we additionally assume that not all muscle fibres are recruited in control conditions, thus 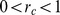. We set 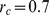 for all muscles. Unfortunately, there are no experimental data that would reveal the correct value of 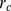. Hence, the value chosen for it remains somewhat arbitrary. In some muscles, there are a few motor units, only. The recruitment is, accordingly, rather coarse. In the muscle model, that means that 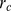 can assume a few discrete values, only, between 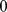 and 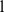. The number of the discrete values 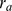 can assume is equal to the number of motor units in the muscle. This means that the number of motor units determines the number of intermediate positions the femur (limb) can attain.

## Results

### Angular movement and network activity during forward stepping

**Figure 4:**
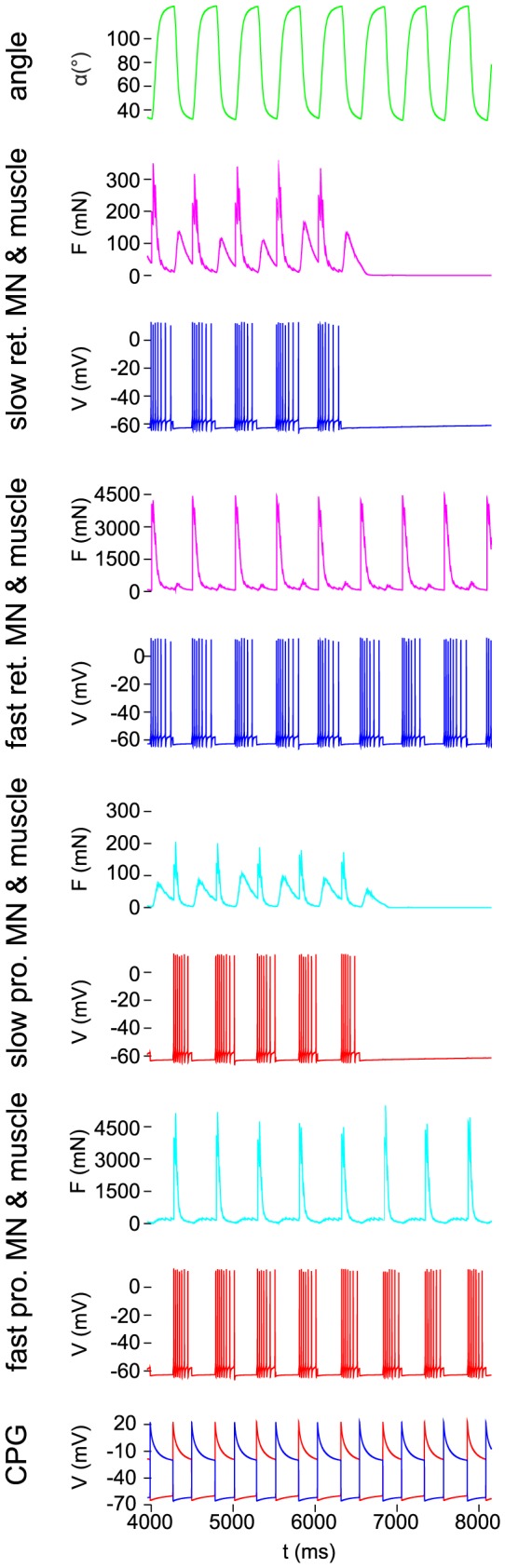
Angular movement and motoneuron and CPG activity in the protractor-retractor neuro-muscular system during forward stepping. The panels show the time evolution of the quantities indicated at the panels. Slow and fast relate to motoneurons and corresponding muscles they innervate. The small and wide peaks between the larger and narrower ones in the activity of the slow muscles are generated by the contraction of the antagonistic muscles (passive stretching). The colour code of the activity of the CPG neurons signalizes the association with the motoneuron type. Note that at 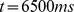, the activity of the slow motoneurons was blocked.

First, the electrical activity of the CPG and the MNs during forward stepping, as well as the resulting angular movement of the femur were simulated. We tested the cases when both slow and fast MNs were simultaneously active (co-contraction of slow and fast muscle fibres) and when the activity of the slow MNs was blocked. The former case roughly corresponds to fast walking of the animal, whereas the latter one is admittedly rather artificial, since the slow MNs are normally recruited earlier during walking than the fast MNs [18]. The results are shown in Fig. 4. The rhythmic co-contraction of the slow and fast muscle fibres of the same muscle (protractor or retractor), as illustrated in panels 2,4 and 6,8 from the top of Fig. 4, respectively, moves the femur alternately forward or backward in the range between 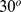 and 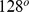 (Fig. 4, top panel) in agreement with experimental data from the stick insect. Fig. 4 also displays the phase relations between CPG activity, MN discharge, muscle contraction, and angular movement during stepping. The activities of the slow and fast MNs innervating the same muscle type (protractor or rectractor) are, up to 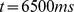, in full synchrony (Fig. 4, panels 2–5, and 6–9 from the top, respectively). At 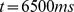, the activity of both the slow protractor and retractor MNs was blocked. The blockade completely abolished the forces in the slow muscles just after a short while (Fig. 4 panels 2 and 6 from the top). It had, however, a negligible effect, only on the angular movement, hardly discernible in Fig. 4. We can therefore conclude that, in the model, the large angular movements during forward stepping are determined by the contractions of the fast muscle fibres of the two muscle types (protractor and retractor). Consequently, if the fast muscle fibres are active, the omission of the slow muscle fibres when modelling angular movements during forward stepping will not lead to discernible simulation error.

### Angular movement and network activity when the fast MNs are inhibited

**Figure 5:**
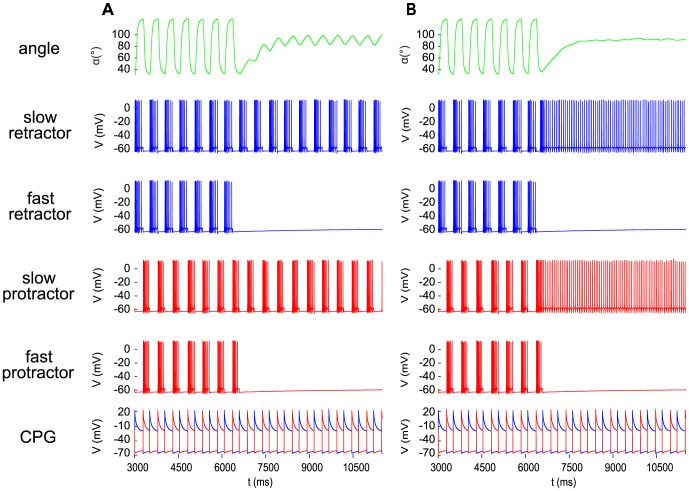
The effect of inhibition of the fast motoneurons. The angular movement and the neuronal activities displayed in the panels are the same as in Fig. 4. Inhibition was brought about in the model by reducing the value of both conductances 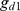 and 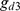 of the inhibitory currents to the interneurons IN1 and IN3, respectively (cf. Fig. 1), from 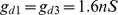 to 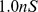 A: leaving the values of the conductances 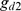 and 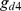 at IN2 and IN4, respectively, unchanged at 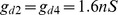 (no change to the input of the slow MNs); B: increasing the values of these conductances to 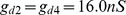 (disinhibition of the slow MNs). Note the large difference in the amplitude of the oscillation after the onset (at 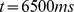) of the inhibition to the fast MNs.

Next we investigated the case when one or both fast MNs were inhibited, i.e. the conductances 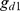 or 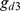, or both of the inhibitory inputs to IN1 and IN3 were strongly reduced. Fig. 5 shows the effect of the inhibition of both fast MNs. The amplitude of the angular movement strongly decreased after the onset of the inhibition (at 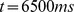). The new range of the angle 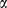 became 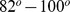, a peak-to-peak amplitude of 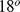. (Compare it to the peak-to-peak amplitude of about 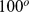 of the angular movement during stepping in Fig. 5A). This is a sign that the contraction kinetics of the slow muscle fibres cannot follow the relatively high frequency 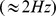 of the driving CPG. The decrease in the amplitude was even more striking if at the same time when the fast MNs were inhibited, the slow ones were activated by disinhibition, i.e. by strongly increasing the inhibition to the INs IN2 and IN4 (Fig. 5B). As a result, the peak-to-peak amplitude was reduced to about 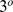. In both cases, the baseline of the small-amplitude oscillation lay at about 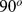. In an earlier model with no slow muscle fibres [19], stationary states of the retraction position of the femur emerged when both (fast) protractor and (fast) retractor MNs were inhibited. This entailed that the forces in both the protractor and the retractor muscle vanished. In this way, a stationary position at 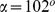 could be attained but the emerging stationary position was, in contrast to the present version of the model, prone to even small perturbations because of lack of stiffness in the muscles. Moreover, the stationary position in the earlier model heavily depended on the phase of the duty cycle in which the MN activity was stopped.

It is a quite important point to bear in mind that the diminution of the small amplitude to an almost negligible value was produced in the present model by the strong simultaneous activity of the slow MNs, hence by sustained co-contractions of the slow muscle fibres in agreement with the experimental results [12]. This mechanism turns out to be biologically more relevant and is in stark contrast to the behaviour of our previous model [19] in which the stationary position was due to the lack of muscle forces.

**Figure 6:**
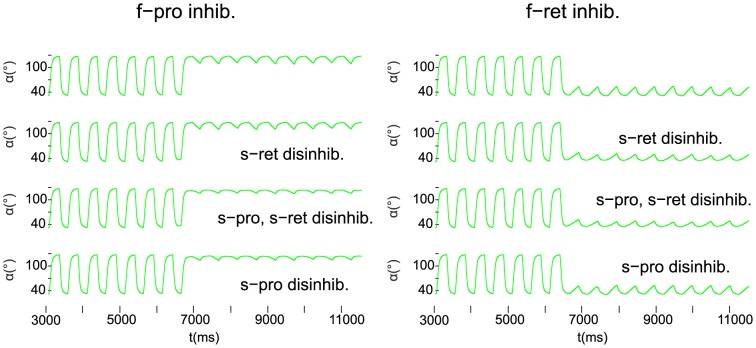
Selective inhibition of the fast motoneurons. Left column: only the fast protractor motoneuron is inhibited. Right column: only the fast retractor motoneuron is inhibited. In both columns: top panel: normal disinhibition of both slow motoneurons (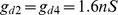); 2nd panel: strong disinhibition of the slow retractor motoneuron, only (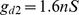, 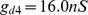); 3rd panel: strong disinhibition of both slow motoneurons (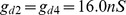); bottom panel: strong disinhibition of the slow protractor motoneuron, only (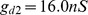, 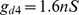). Note that the small-amplitude oscillation due to the activity of the slow motoneurons is positioned close to the extremal angle corresponding to the *uninhibited* fast motoneuron, e.g. to the maximal retraction position if only the fast retractor motoneuron remains active (left column).

When only one of the fast MNs was inhibited and the other kept on firing, the small-amplitude oscillation appeared near one of the extremal values of the angular movements after the onset of the MN inhibition. This is shown in Fig. 6, the inhibition starting at 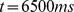 in the simulations. As indicated in Fig. 6, the column on the left comprises the cases when the fast protractor MN was inhibited, the other column those when the fast retractor MN underwent inhibition. Accordingly, the small-amplitude oscillation took place near to the extremal retraction and protraction angle, respectively. The peak-to-peak amplitude of the small-amplitude oscillation varied between 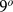 and 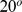 in the former case, and between 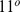 and 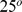 in the latter (Fig. 6). We see thus a certain asymmetry between the two cases. The minimum of the peak-to-peak amplitude of the small-amplitude oscillation was attained in both groups of cases when both slow MNs were strongly disinhibited (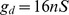), (i.e. activated). When only one of the slow MNs were inhibited, the size of the amplitude of the small-amplitude oscillation depended on which of the slow MNs was inhibited. For example, in the left column of Fig. 6 (inhibition of the fast protractor MN), the peak-to-peak amplitude stayed close to its minimum (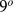) when the slow protractor MN was strongly disinhibited, i.e. activated, (row 3 and 4 in the left column of Fig. 6). Otherwise, the peak-to-peak amplitude was close to that in the case when both slow MNs received normal activation (disinhibition) (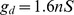). Analogously, the peak-to-peak amplitude of the small-amplitude oscillation was near to its minimum (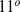) when the slow retractor MN was strongly activated (disinhibited) (row 2 and 3 in the right column of Fig. 6). It is an interesting property of our model that in both cases, the inhibition of the fast MN of a specific type and strong activation (disinhibition) of the *same* type of slow MN produced smaller amplitudes than when the slow MN of the antagonistic type was activated (disinhibited).

In summary, we found that the small-amplitude oscillation of the angle 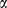 driven by the slow MNs can be positioned near to one of the extremal angles if only one of the fast MNs is inhibited. Again, the peak-to-peak amplitude of this oscillation depends on the degree of activation (disinhibition) of the slow MNs.

### Producing static protraction-retraction positions in the model by using recruitment of the muscle fibres

In the protractor-retractor muscle system, the pool of the protractor MNs consists of about 17, that of the retractor ones of about 25 MNs [20]. Hence our model assumptions concerning the recruitment of muscle fibres appear to be acceptable in this case. There are, however, no data on the proportion of MNs supplying fast or slow muscle fibres. We assumed that, in the model, there were equally many (10) fast and slow MNs in each MN pool. This allows a resolution of 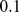 of the recruitment levels (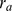 and 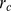 in eqn. 3). Moreover, we chose 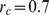 for both the protractor and retractor muscles (cf. Methods). That is, during stepping, 7, out of the 10 motor units, are assumed to be recruited, hence involved in producing muscle force in both of the antagonistic muscle pairs during stepping. We have made this assumption because it seems physiologically unreasonable that during normal locomotion, i.e. when the animal is not exposed to any additional load, only to its own weight, all muscle fibres would be recruited. On the other hand, during locomotion, the muscles of the legs move the mass of the whole body. One could therefore reckon with a substantial degree of recruitment of muscle fibres in this condition. Investigations in the locust [21] showed that the locust does have “recruitment reserve” when horizontally walking, since at vertical climbing, additional protractor-retractor muscles are recruited. Hence, the control recruitment level of 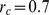 does not appear unrealistic.

**Figure 7:**
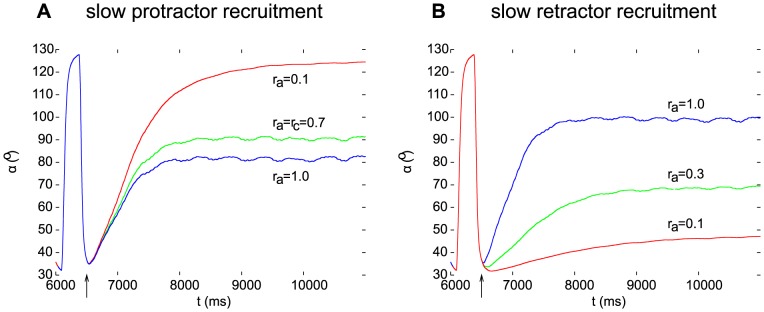
Steady states and recruitment levels. Different steady states are brought about by different recruitment levels of the slow muscle fibres in the protractor (A) or retractor (B) muscle. The actual recruitment levels are indicated in the panels next to the individual curves. All fast motoneurons were inhibited in these simulations. The arrows mark the start of the inhibition of the fast motoneurons (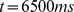).

Next we carried out simulations using various recruitment levels of the slow and fast muscle fibres in order to study the role muscle recruitment plays in shaping the mechanical movement of a limb. To be more precise, the fast MNs of both muscle types were first inhibited. Thus only the slow muscle fibres showed activity at a number of recruitment levels. We found that the relation between the recruitment levels in the antagonistic muscles determined the stationary retraction position of the femur. Fig. 7 illustrates this for a number of positions. Fig. 7A shows the results when only the recruitment level of muscle fibres in the slow protractor muscle was varied, but the recruitment level in the retractor muscle was kept at its control value (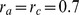). In Fig. 7B, the situation is analogous to that in Fig. 7A, only the roles of the muscles are exchanged. It can be seen that by suitable choices of the recruitment levels (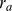 values) in the protractor and retractor muscles, a steady state at any angle 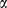 within the range 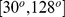 can be attained within the error limits due to the finite (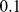) resolution of the muscle fibre recruitment. One can thus conclude that the slow antagonistic muscle fibres can be driven by the associated neuronal network so as to keep the femur in any desired steady-state position.

**Figure 8:**
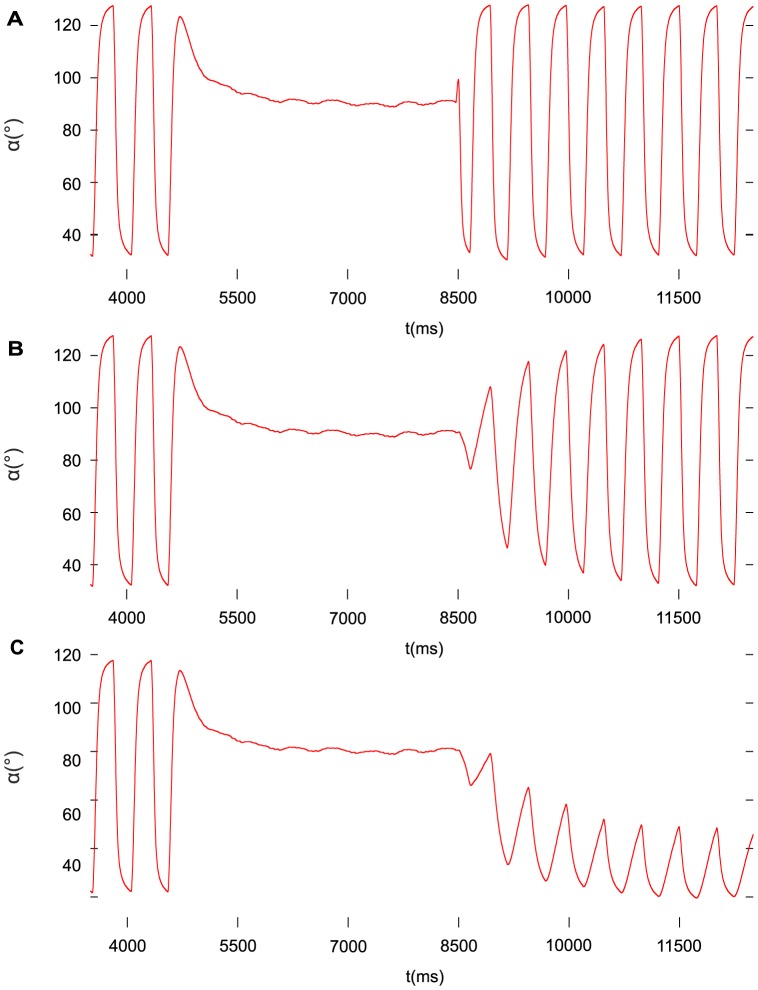
Restoring normal stepping from steady state (standing still). A: instantaneously, B: gradually, C: partially. At 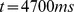, the fast motoneurons are inhibited, and the slow ones disinhibited, hence the steady state. At the same time, the recruitment of the fast fibres decreases to virtually zero, whereas that of the slow ones remains unchanged. At 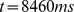, the inhibition of the fast motoneurons and the disinhibition of the slow motoneurons are stopped, and, in A and B, the recruitment of the fast protractor and retractor muscle fibres starts, while in C, only the fast protractor muscle fibres are recruited. The recruitment plays a crucial part in the restoration process. In case A, the recruitment of the fast fibres occurs very fast (instantaneously), while in the cases B and C, it does so linearly over a time interval of 3 s. Note, however, that in B, the increase of the amplitude of the angular movement 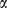 is nonlinear. In C, the full amplitude of the angular movement 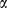 is not restored, because the fast retractor muscle fibres are not recruited.

In another series of simulations, we aimed at emulating the transition from steady state to rhythmic stepping (locomotion) by using increasing recruitment in time of the fast muscle fibres, while the slow ones remained at their normal (control) recruitment level. Fig. 8 shows the results of these simulations. We considered three cases: very fast (instantaneous) (Fig. 8A), gradual (Fig. 8B), and partial restoration of the stepping movement (Fig. 8C). In the first case, the recruitment of both the fast protractor and retractor muscle fibres occurred very fast, implemented by a pulse (jump) time function (Fig. 8A), whereas in the second case, ramp time functions were used to control the actual level of recruitment of the fast muscle fibres (Fig. 8B). Note that although the fast, both protractor and retractor, muscle fibres had linearly increasing recruitment in time, the amplitude of the angular movement 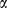 of the femur was increasing nonlinearly (Fig. 8B). Finally, Fig. 8C shows a case of partial restoration of the protraction-retraction angular movement during stepping. Here, no recruitment of the fast retractor muscles took place, while the fast protractor ones were gradually recruited. As a result, the full range of the angular movement was not restored, and the angular oscillation remained near the extremal protraction position, and had a reduced peak-to-peak amplitude. A later recruitment of the fast retractor muscle fibres would have restored the full range of the angular movement during stepping (not illustrated).

### Muscle forces during inhibition of the fast MNs

**Figure 9:**
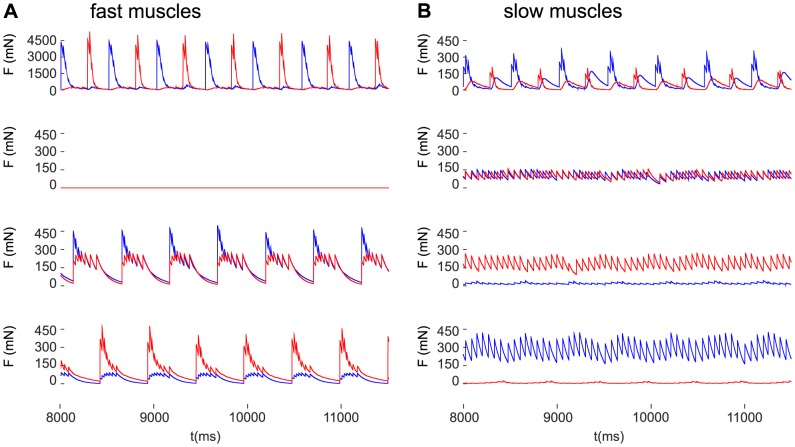
Muscle forces in control conditions and during inhibition of one or both fast motoneurons. A: forces in the fast protractor (red) and retractor (blue) muscles. B: forces in the slow protractor (red) and retractor (blue) muscles. Top row of panels: control condition when both the fast and the slow muscles are active; 2nd row of panels: both fast motoneurons are inhibited (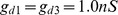); 3rd row of panels: only the fast protractor motoneuron is inhibited (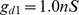, 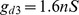); bottom row of panels: only the fast retractor motoneuron is inhibited (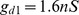, 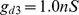).

It is of considerable interest to find out what the acting muscle forces are during the small-amplitude oscillation, or in a nearly static state, when one or both fast MNs are inhibited. The muscle forces produced by the model in these conditions are shown in Fig. 9 together with the control case, i.e. when both slow and fast muscles are active.

In control conditions (i.e. during stepping), the antagonistic fast muscles contract alternately, with negligible co-contraction (Fig. 9A, top panel). In the slow muscles, co-contraction is discernible but the alternate contractions are still dominant (Fig. 9B, top panel). This situation is also depicted in Fig. 4, although in a different context. When both fast muscles are inhibited, the contraction forces of both fast muscles vanish in the model (Fig. 9A, second panel). The reason for this is that there is no explicit passive elastic component in our muscle model, hence the variable elasticity modulus 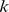 eventually decays to zero if there is no drive from the MNs of an antagonistic muscle pair (see eqn.s 1 and 2). However, there are still antagonistic forces of the slow muscle fibres present (Fig. 9B, second panel). They are, in the present case, almost exactly equal, since the steady state is around 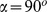, i.e. both types of slow muscle fibres are at their control level of recruitment (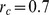). If only one of the fast muscles is inhibited, the other, the active one evokes co-contraction of the antagonistic fast muscle, even with no MN drive to the inhibited one (Fig. 9A, the two bottom panels). This apparent passive force arises because at the switch of direction of movement of the femur stationary conditions (vanishing angular velocity and acceleration) have to be fulfilled. These conditions impose constraints on the stationary values of the spring constants (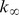 values in eqn. 1) of an antagonistic muscle pair (see Methods and, for a detailed explanation, [14]). Hence, neither of the spring constants will be zero.

As for the slow muscles, the force in the slow muscle whose fast counterpart is inhibited is much larger than the force in its antagonistic counterpart, compensating for the lack of force in the corresponding fast muscle (Fig. 9B, the two bottom panels). For example, the bottom panels of Fig. 9 show the case when the fast retractor muscle is inhibited. Accordingly, the fast muscles show co-contraction, with the force of the retractor muscle (blue) being much smaller than that of the fast protractor muscle (red) (Fig. 9A, bottom panel). At the same time, the force of the slow retractor muscle (blue) is much larger than that of the slow protractor muscle (red) (Fig. 9B, bottom panel).

## Discussion

In this study, we investigated, by means of a neuro-mechanical model, possible mechanisms that can produce and maintain steady-state positions of limb joints, as well as the transition between angular movements during stepping and the above positions. The mechanisms suggested can serve as elements of more complex neuro-muscular coordination which produces stable postures or aimed movements in animals, in our specific case, in the stick insect. The model we used is an extension of the one by [14] and includes separate slow and fast muscle fibres and their dedicated neuronal control network. One should, however, bear in mind that this is a strong simplification of the real situation. The separation between slow and fast muscle fibres is not as sharp in the animals as we implemented it in our model. Rather, experimental data indicate a gradual transition between fast and slow muscle fibres, i.e. a continuum of muscle properties [4], [7]. We, for the sake of simplicity, ‘discretized’ this continuum by dividing the muscle fibres into just two groups of contrasting properties. In addition, we implemented the differential recruitment of muscle fibres. As far as we are aware of, this is the first model for insects that possesses such properties. Previous muscle models were more specialized. They either did not take the differing properties of the slow and fast muscle fibres into account, e.g. [22]–[25], or did not include the dedicated neuronal control network of the muscles [26], [27]. They concentrated on producing specific muscle behaviour using certain aspects of the muscle function. In contrast, our integrated model enables us to study a more complex phenomenon: the functional differentiation between static and dynamic aspects of movement control.

We constructed this extended model only for the protractor-retractor neuro-muscular system as it plays an important part in maintaining posture, i.e. when an insect is standing still, or when the joint is fixed during sideward stepping. However, the same type of extension can be done, with some constraints due to the much fewer motor units, for the other neuro-muscular systems (joints), too. Indeed, a model that has fast and slow muscle fibres in each of the three pairs of main leg muscles complete with their neuronal control (MNs) will be introduced in the accompanying paper (Toth et al., unpublished results). Simulations with the extended model, presented in this paper, highlighted some model properties that might be of physiological relevance in insects, or, possibly in other animals, too. First of all, we found in the simulations that during (fast) stepping (also regarded as control conditions in this study), the fast muscles determine the stepping, more precisely, the protraction and retraction movement. The effect of the slow muscle fibres in this case is minute. Although there exists no direct experimental evidence for this simulation result, indirect evidence can be derived from [18], [12], and [17]. In [18], higher treadwheel velocities led to recruitment of fast MNs resulting in large depolarizations in the fast fibres of the stick insect's flexor tibiae muscle. Similarly, [12] showed that the fast MN of the extensor tibiae muscle of the stick insect produced much larger depolarizations in the muscle fibres it innervated than the slow MN driving the slow muscle fibres. In the experiments in [17], additional stimulation of the slow extensor MN (SETi) did not result in a discernible increase of the muscle force due to the activity of the fast extensor MN (FETi). The simulations further showed that when the MNs that innervate the fast muscle fibres (fast MNs) are inhibited but the slow MNs are not, the angular movement becomes a small-amplitude oscillation about the angle 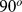. By further disinhibition (activation) of the slow MNs, the amplitude of this oscillation can be reduced to become negligible. However, the nearly steady state, too, represented by the small-amplitude oscillation in the model, has its experimental analogue: the extremities of a standing stick insect do not remain in a strictly static position but show slight rocking (oscillatory) movements [28]. This small-amplitude oscillation most likely occurs because the slow inherent contraction kinetics of the slow muscle fibres cannot cope with the relatively high frequency 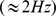 of the CPG that ultimately drives these fibres. Most importantly, [28] found that rocking was produced by the slow muscle fibres, only, and that the frequency of rocking was about 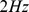. This is in excellent agreement with the properties of our model and the simulation results.

Taking, in addition, the gradual recruitment of the muscle fibres into account in the model, the steady state of the protractor-rectractor angle 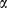 can be set at any position within the angular range of 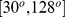. The simulation results produced by the model are in agreement with the important experimental finding that steady states are maintained by co-contraction of antagonistic (slow) muscle pairs, and not because of the lack of muscle forces [12], [25]. Co-activation of the MNs driving the antagonistic slow muscles can improve the steady state, i.e. reduce the amplitude of the remaining small-amplitude oscillation to a negligible size. In Table 1, the locomotion status of a leg and the functional modes of the MNs that produce this status are summarized. Table 2, in turn, lists the physiological functions that shape the properties of the angular movement. Clearly, the frequency of the rhythmic angular movement (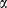) is determined by that of the CPG. The amplitude of the oscillation depends on whether the fast MNs are active at all, and if so, how many fast motor units are actually recruited (recruitment). The average position of the oscillation, or the static position is mainly determined by the activity of the slow MNs and the recruitment of the slow motor units but partial recruitment of the fast muscle fibres can also make a substantial contribution (cf. Fig. 6). Our model suggests that the steady-state position is more likely maintained by the recruitment ratio of the slow fibres in the antagonistic (protractor-retractor) muscle pair, than by the forces in the individual muscle fibres.

**Table 1:** Locomotion status and motoneuron (MN) activity that produces it.

Locomotion status	MN type	
	fast	slow
normal stepping	+	(+)
small-amplitude oscillation	−	+
(near) steady state	−	++

“
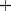
”: excited, “
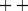
”: strongly excited (disinhibited), “
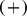
”: activated but not effective, “
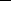
”: inhibited.

**Table 2:** The physiological functions responsible for changing the attributes of the angular movement.

Angular movement	MN activity	Muscle recruitment
amplitude	fast	fast
average position	slow (+fast)	slow (+fast)
frequency	CPG	

Fast and slow relate to fast and slow muscle fibres or motoneurons (MNs), respectively. (+fast) means that additional activity of the fast muscle fibres (or MNs) substantially contributes to producing the average position of the protractor-retractor angle 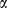 during oscillation.

The transition between the angular movement during stepping and steady-state position could also be simulated by the model. Here, the time-dependent recruitment levels of the *fast* muscle fibres play a crucial part in the transition process. Depending on the dynamics of the recruitment changes, the transition can take different shapes. The movements simulated here can be regarded as elements of more complex movements of the limbs in which, of course, several joints will be involved [29]–[31]. In the accompanying paper (Toth et al., unpublished results), we shall present simulation results on the intra-leg coordination of the activities of the three main leg muscle pairs.

Our model provides information on the muscle forces that arise in different conditions (Fig. 9). While in the majority of conditions, the size of the forces in the slow and fast muscle fibres seem to be reasonable, the forces generated by the fast muscle fibres of the model in control conditions (normal stepping) appear to be somewhat too large. Since there do not exist direct force measurements in the protractor or retractor muscles, it is hard to judge the possible error occurring in the model in this condition. However, when we applied the same muscle model, with suitable numerical values of the parameters to the extensor and flexor muscles, in which force measurement were made [17], we found comparable muscle forces in experiment and simulation (data not shown).

Irrespective of this problem, our model makes interesting predictions with regard to the muscle forces arising in various conditions, e.g. during stepping, or in (near) steady state. The model predicts that during stepping, the alternate contraction of the antagonistic muscles, protractor and retractor, is dominant in both the fast and the slow muscle fibres. These forces are generated by the excitatory drive by the corresponding MNs. In (near) steady state, however, when the fast MNs are inhibited, only the slow muscle fibres stay active, the fast ones do not contribute to the total muscle force. The forces in the slow muscle fibres are generated actively by the excitatory drive, occasional co-activation, of the slow MNs. There is some experimental evidence, although in the extensor tibiae muscle of the stick insect [7], [12], that fast muscle fibres do not develop substantial force during steady state and it is the slow muscle fibres that maintain this state. It is also predicted by the model that when only either the fast protractor or fast retractor muscle fibres are inhibited, co-contraction becomes dominant in the fast muscle fibres. The contraction in the fast muscle fibres that do not receive MN activity is due to the nonlinear elasticity of the muscle represented by the, in this case, non-vanishing spring constant that produces an apparently passive component of the muscle function. The slow muscle fibres will perform co-contractions, only if their respective MNs are co-activated. The force in those slow muscle fibres whose fast counterparts are inhibited (e.g. retractor in the bottom panel of Fig. 9B) is now much larger than in the antagonistic slow fibres. Thus the force produced by the slow fibres, to some extent, compensates for the lack of force in the corresponding fast fibres.

Some of these properties can be deduced from the way our muscle model has been constructed. Contrary to some other muscle models (e.g. [22]–[25]), it does not have passive elastic elements. The variable spring constants will therefore tend to zero, if *neither* of the antagonistic muscles receives any excitatory signals from their corresponding MN. However, if one of the muscles receives electrical excitation from its MN, *neither* of the spring constants will vanish. This seems reasonable, since the active muscle, with MN driving, stretches its antagonist evoking elastic contraction force in it. Thus our muscle model is capable of producing apparently passive (elastic) contraction force in the muscle with no MN drive. The underlying property of the model which results in this behaviour is that the spring constants of the antagonistic muscles are not independent of each other. They are subject to a constraint that arises from the switch conditions from protraction to retraction and *vice versa* (cf. Methods and Results and [14]). But note that the muscle fibres in our model do not possess residual stiffness.

Closely related to this fact is that our neuro-muscular model does not include the common inhibitor MNs, either. Slow muscle fibres, but no fast ones, are innervated by them in the stick insect and locust [7], [12] and also in the cockroach [32]. Their physiological role is to remove the residual stiffness of the slow muscle fibres during locomotion (stepping) in order to improve the dynamics of the movement [12], [33]–[35]. The common inhibitory MN CI1 acts on all main leg muscles, except for the m. flexor tibiae, which receives input from the inhibitory MNs CI2 and CI3. We omitted the common inhibitory MNs from the present model, comprising one single antagonistic muscle pair, for the sake of simplicity. CI1 (and CI2–CI3), and the residual stiffness of the slow muscle fibres will become quite important when we deal with intra-leg coordination of the activities of the three pairs of antagonistic leg muscles (cf. accompanying paper, Toth et al., unpublished results).

Our simulation results highlight the differing roles of the fast and slow muscles during locomotion and maintaining body posture. They receive support from experimental findings. [7] showed the spatial separation of slow and fast muscle fibres in the extensor muscle. Here, however, it should again be remembered that the strict classification into slow and fast muscle fibres is a simplification in the model. Even though there are no direct data from the protractor-retractor muscles, we would assume to find a similar situation there, as well. Furthermore, [12] found differing functions of the slow and fast muscle fibres in the stick insect's extensor muscle similar to those observed in the simulations. Again, these experimental findings relate to the extensor muscle but there is recent evidence [13] that slow and fast muscle fibres do exist in both the protractor and the retractor muscle of the stick insect. We suggest that they share functional roles similar to those in the extensor muscle as observed in the experiments [12], and to those produced by the simulations with the extended neuro-muscular model. As a general tool, selective blockade or (partial) elimination of the fast or slow muscle fibres from the protractor or rectractor muscle in experiments could help test the predictions of our model.

## Supporting Information

Appendix S1 **A summary of the neuron models used in the neuro-muscular model of the paper.**
(PDF)

Appendix S2 **A summary of the quantitative description of the mechanical motion of the femur governed by the muscle forces of the protractor and retractor coxae muscles.**
(PDF)
