# Supplementary material for: A Neuro-Mechanical Model of a Single Leg Joint Highlighting the Basic Physiological Role of Fast and Slow Muscle Fibres of an Insect Muscle System
Source: PLoS One. 2013 Nov 11;8(11):e78247. doi: 10.1371/journal.pone.0078247 (PMC3823925; doi:10.1371/journal.pone.0078247)
Supplement: Appendix S1 — A summary of the neuron models used in the neuro-muscular model of the paper. (PDF) [file pone.0078247.s001.pdf]

Appendix S1: Equations of the neuron models

CPG-neurons and interneurons

The constituent currents are

I_{NaP}: slowly inactivating Na- current;

I_L: leakage current;

I_{syn}: synaptic current from other neurons;

I_{app}: input current (e.g. from sense organs or from the brain).

The model equations read:

$$\begin{aligned} C_m dV/dt &= -(I_{NaP} + I_L + I_{syn} + I_{app}) \quad \text{with} && (1)\\ I_{NaP} &= g_{NaP} m_\infty(V) h (V - E_{Na}) && (2)\\ I_L &= g_L(V - E_L) && (3)\\ I_{syn} &= g_{syn} s_\infty(V_{syn})(V - E_{syn}) && (4)\\ I_{app} &= g_{app}(V - E_{app}) \quad \text{and} && (5)\\ dh/dt &= (h_\infty(V) - h)/\tau_h(V). && (6) \end{aligned}$$

The membrane potential $V(t)$ and the inactivation variable $h(t)$ of I_{NaP} are the model variables, both are functions of time. The function $m_\infty(V)$ is the steady-state value at V of the activation variable m of I_{NaP}, and $h_\infty(V)$ is the steady-state value (at V) of h. In eqn. 4, the function $s_\infty(V_{syn})$ is the actual value of the synaptic activation induced by *another* cell with membrane potential V_{syn}. All of these voltage functions have the same form:

$$z_\infty(V) = 1/(1 + \exp(\gamma_z(V - V_{hz}))) \qquad (7)$$

where $z = m, h, s$, while the formula for 'time constant' $\tau_h(V)$ reads

$$\tau_h(V) = \frac{1}{\epsilon} \frac{1}{\cosh(\gamma_\tau(V - V_{\tau h}))}. \qquad (8)$$

The coefficients g_x in eqns. 2-5 are the maximal conductances of the membrane currents, and the parameters E_x the corresponding reversal potentials; C_m is the membrane capacitance.

Motoneurons

The constituent currents are:

I_{Na}: fast inactivating Na-current of the action potential;

I_K: delayed rectifier K-current of the action potential;

I_q: 'adaptation' (K-)current;

I_L: leakage current;

I_{syn}: synaptic current from other neurons;

I_{app}: input current (e.g. from sense organs or from the brain).

The current balance equation reads:

$$C_m dV/dt = -(I_{Na} + I_K + I_q + I_L + I_{syn} + I_{app}) \tag{9}$$

The individual membrane currents are of the form:

$$I_x = g_x m_x^p h_x (V - E_x). \tag{10}$$

where $x = Na, K, q, L$ (I_{syn} and I_{app} are of the same form as in the CPG neuron model: eqns. 4, 5). The parameters g_x and E_x have the same meaning as before (see preceding section). The parameter p is an integer. The (in)activation variables h_x and m_x, respectively, obey a differential equation of the form:

$$dy/dt = \alpha_y(V)(1 - y) - \beta_y(V)y \tag{11}$$

where y stands for h_x or m_x. The coefficients of eqn. 11 are nonlinear functions of the membrane potential V. They can have different forms depending on the type of the membrane current. The specific $\alpha(V)$ and $\beta(V)$ functions are listed below for each individual current type.

I_{Na} current

m_{Na}:

$$\alpha_{m_{Na}}(V) = \frac{a_{m1}(a_{m2}-V)}{\exp(a_{m3}(a_{m2}-V))-1} \quad (12)$$

$$\beta_{m_{Na}}(V) = \frac{b_{m1}(b_{m2}-V)}{\exp(b_{m3}(b_{m2}-V))-1} \quad (13)$$

h_{Na}:

$$\alpha_{h_{Na}}(V) = a_{h1}\exp(a_{h3}(a_{h2}-V)) \quad (14)$$

$$\beta_{h_{Na}}(V) = \frac{b_{h1}}{\exp(b_{h3}(b_{h2}-V))+1} \quad (15)$$

I_K current

m_K:

$$\alpha_{m_K}(V) = \frac{a_{m1}(a_{m2}-V)}{\exp(a_{m3}(a_{m2}-V))-1} \quad (16)$$

$$\beta_{m_K}(V) = b_{m1}\exp(b_{m3}(b_{m2}-V)) \quad (17)$$

Adaptation current I_q

$$m_{q\infty} = \frac{1}{1+\exp(s_q(V-V_{hq}))} \quad (18)$$

$$r_q = const. \quad (19)$$

$$\alpha_{m_K}(V) = m_{q\infty}r_q \quad (20)$$

$$\beta_{m_K}(V) = (1-m_{q\infty})r_q \quad (21)$$

where $m_{q\infty}$ is the steady-state value of m_q at a given value of V and r_q is the rate constant. For further details see [1, 2].

References

1. Daun S, Rybak IA, Rubin J (2009) The response of a half-center oscillator to external drive depends on the intrinsic dynamics of its components: a mechanistic analysis. J Comput Neurosci 27: 3-36.

2. Daun-Gruhn S, Toth TI (2011) An inter-segmental network model and its use in elucidating gait-switches in the stick insect. J Comput Neurosci 31: 43-60.
