# Supplementary material for: A Neuro-Mechanical Model of a Single Leg Joint Highlighting the Basic Physiological Role of Fast and Slow Muscle Fibres of an Insect Muscle System
Source: PLoS One. 2013 Nov 11;8(11):e78247. doi: 10.1371/journal.pone.0078247 (PMC3823925; doi:10.1371/journal.pone.0078247)
Supplement: Appendix S2 — A summary of the quantitative description of the mechanical motion of the femur governed by the muscle forces of the protractor and retractor coxae muscles. (PDF) [file pone.0078247.s002.pdf]

Appendix S2: Equation of mechanical motion of the femur

The equation of mechanical motion of the femur under the influence of the protractor and retractor muscles reads:

$$\ddot{\alpha} = c_4 \sin\alpha \left(\frac{F_R}{l_R} - \frac{F_P}{l_P} \right) - c_5 \dot{\alpha} \tag{1}$$

In this eqn., $\alpha(t)$ is the retraction angle of the femur, l_P and l_R are the actual lengths of the protractor and retractor muscle, respectively. F_P and F_R are contraction forces in the protractor and retractor muscle, respectively. c_4 and c_5 are constants with c_5 expressing the extent of damping (viscosity) in the system. The contraction forces F_P and F_R are nonlinear elastic forces of the form [1]:

$$F_P = k_P(l_P - l_{Pmin})^2 \tag{2}$$

$$F_R = k_R(l_R - l_{Rmin})^2 \tag{3}$$

where k_P and k_R are the spring constants, and l_{Pmin} and l_{Rmin} the minimal lengths of the respective muscles. For a detailed explanation see [2].

References

1. Guschlbauer Ch, Scharstein H, Büschges A (2007) The extensor tibiae muscle of the stick insect: biomechanical properties of an insect walking leg muscle. J Exp Biol 210: 1092-1108.

2. Tóth TI, Knops S, Gruhn S (2012) A neuro-mechanical model explaining forward and backward stepping in the stick insect. J Neurophysiol 107: 3267-3280.
